# Excessive nitrogen application dampens antioxidant capacity and grain filling in wheat as revealed by metabolic and physiological analyses

**DOI:** 10.1038/srep43363

**Published:** 2017-02-24

**Authors:** Lingan Kong, Yan Xie, Ling Hu, Jisheng Si, Zongshuai Wang

**Affiliations:** 1 Crop Research Institute, Shandong Academy of Agricultural Sciences, Jinan 250100, China; 2 College of Life Science, Shandong Normal University, Jinan 250014, China

## Abstract

In this study, field-grown wheat (*Triticum aestivum* L.) was treated with normal (Nn) and excessive (Ne) levels of fertilizer N. Results showed that Ne depressed the activity of superoxide dismutase and peroxidase and increased the accumulation of reactive oxygen species (ROS) and malondialdehyde. The normalized difference vegetation index (NDVI) was higher under Ne at anthesis and medium milk but similar at the early dough stage and significantly lower at the hard dough stage than that under Nn. The metabolomics analysis of the leaf responses to Ne during grain filling showed 99 metabolites that were different between Ne and Nn treatments, including phenolic and flavonoid compounds, amino acids, organic acids and lipids, which are primarily involved in ROS scavenging, N metabolism, heat stress adaptation and disease resistance. Organic carbon (C) and total N contents were affected by the Ne treatment, with lower C/N ratios developing after medium milk. Ultimately, grain yields decreased with Ne. Based on these data, compared with the normal N fertilizer treatment, we concluded that excessive N application decreased the ability to scavenge ROS, increased lipid peroxidation and caused significant metabolic changes disturbing N metabolism, secondary metabolism and lipid metabolism, which led to reduced grain filling in wheat.

Nitrogen (N) is one of the major nutritional elements of wheat (*Triticum aestivum* L.), and in modern agriculture, N fertilizers are widely used to nourish plants, increase yield and improve end-use quality. Optimal N nutrition is fundamental to the growth and productivity of cereal crops. However, farmers in many parts of the world prefer to apply N fertilizer in excess in attempts to increase the yield. China is a major user of N fertilizers and accounts for 40% of the total global use since 2006^1^. As a consequence of excessive fertilizer use, superfluous N is lost from the plant-soil system, causing environmental damage, and lodging is a very common problem due to large populations and the inhibition of K^+^uptake^2^. Simultaneously, increases in the N supply promote the development of some pathogens^3^, affect plant disease resistance and increase disease susceptibility, a phenomenon called NIS (Nitrogen-Induced Susceptibility)^4^.

During the grain filling period, leaf senescence occurs, accompanied by the programmed degradation of cell constituents and a burst of reactive oxygen species (ROS), such as H_2_O_2_ and superoxide anion radicals, in the chloroplasts, mitochondria and peroxisomes due to aerobic metabolism in both photosynthetically active and senescent cells^5^,^6^. The toxicity of ROS is controlled by different enzymatic and non-enzymatic antioxidant defenses. Superoxide dismutases (SODs), peroxidases (PODs), catalases and the ascorbate-glutathione cycle enzymes are the primary antioxidant enzymes^6^,^7^. Under stress, this equilibrium is altered, and ROS overproduction leads to the deleterious degradation of stromal proteins such as Rubisco and chloroplastic glutamine synthetase^8^,^9^.

In addition to genetic control, senescence is regulated by joined actions of external (e.g., N availability, light) and internal (e.g., regulating metabolites, C/N ratio) signals^10^,^11^. Sugar regulation of leaf senescence is also dependent on plant N status and soil N content^12^. Therefore, a better understanding of the detrimental effects of excessive N and the synchronization of N application with actual crop demand will offer a new strategy for grain development based on the regulation of programmed senescence.

Metabolite profiling is an important technology that is increasingly used to probe a variety of metabolic events in plants and thus promotes a more comprehensive understanding of physiological responses to variations in biological conditions^13^. Such metabolite profiles provide not only a much broader view for a systematic adjustment in metabolic processes than conventional biochemical approaches but also an opportunity to reveal new insights regarding metabolism. Recently, many metabolomics studies have been conducted in cereal crops such as rice^14^,^15^, maize^16^, barley (*Hordeum vulgare* L.)^17^,^18^, foxtail millet (*Setaria italica* L.)^19^, and bread and durum wheat^20^,^21^. These studies focused primarily on the metabolite profiling of plants subjected to stress such as heat, drought and nutrient deficiency to assess the effects of environmental factors on profile characteristics. This technology offers one perspective and approach to evaluate the metabolomic status of a plant to gain insights into nutrient metabolism and to investigate the mechanisms for the efficient use of nutrients^13^; however, the metabolomic modification of leaves following the application of excessive N has not been investigated in wheat.

Ground-based platform systems are a good tool to monitor and manage crop conditions in precision agriculture and are widely used for monitoring crop conditions. In particular, NDVI is one of the best-known vegetation indices and has been particularly useful in estimating growth and N status, senescence parameters and grain yield in wheat and other cereals^22^.

Despite the agronomic importance of wheat, little experimental information is available for physiological and metabolite changes of the flag leaf (the primary source of assimilates for grain growth) of wheat in response to excessive N during grain development. Providing this information is essential in China because many farmers apply excessive N rather than synchronizing N supply with crop demand^23^,^24^. The development of improved strategies for the efficient management of fertilizer N and the sustainability of crop productivity in the wheat production areas of China are critical. The present study was conducted in a wheat field in northern China, and the objectives were to investigate the effects of excessive N on the differential metabolism and senescence-related physiology of wheat to increase our understanding of the responses of this crop to an environment with excess N.

## Results

### Effects of excessive N on ROS content and cell membrane lipid peroxidation

**Figure 1:**
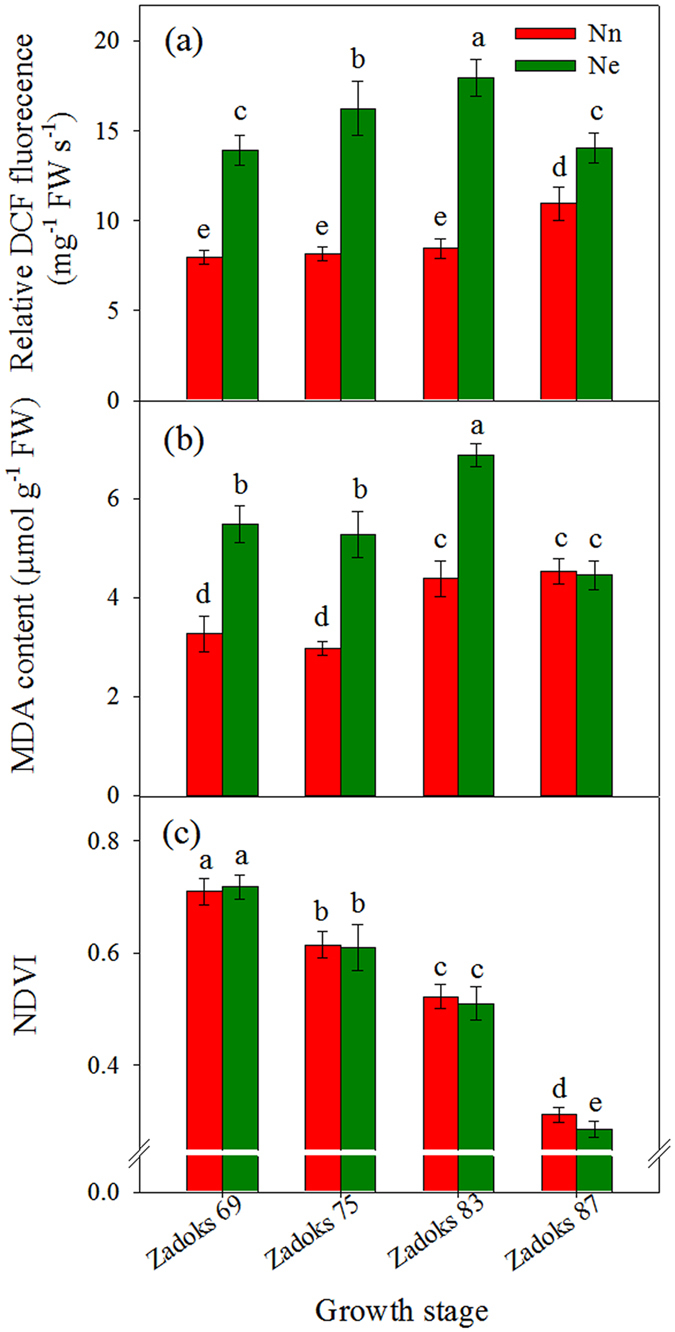
Comparison of the relative DCFH-DA fluorescence intensity (**a**) and MDA (**b**) of flag leaves and the vegetation index measured with a spectroradiometer (NDVI) (**c**) during grain filling of wheat in Nn and Ne treatments. ROS concentration was measured using DCFH-DA, which is oxidized by ROS to DCF. Fluorescence was determined 20 min after the incubation of flag leaf tissues with DCFH-DA. Bars represent the mean ± SD of four replicates. The columns labeled with different letters are significantly different at *p* < 0.05 according to Duncan’s test for multiple comparisons using DPS software.

The ROS relative concentration was evaluated by measuring the fluorescence intensity developing from the oxidation of 2′,7′-Dichlorofluorescein diacetate (DCFH-DA) (Sigma-Aldrich, Germany) in flag leaves of both Nn and Ne treatments. After anthesis (Zadoks 69), ROS increased and peaked at early dough (Zadoks 83) in flag leaves of Ne wheat (Fig. 1), whereas in flag leaves of Nn wheat, a relatively stable ROS level was maintained during the periods of anthesis and early dough, which was followed by a significant increase at maturity stage. Nevertheless, the accumulation of ROS was consistently and significantly higher in Ne than that in Nn plants throughout the grain-filling phase (*p* < 0.05).

Electron microscopy was used to detect H_2_O_2_ in leaf cells using a technique based on the deposition of electron-dense cerium perhydroxides formed by the reaction of cerium ions with H_2_O_2_. In leaves of wheat from both Nn and Ne treatments, precipitates of electron-dense cerium perhydroxides indicated that H_2_O_2_ was located predominantly within the cell walls and the cell membrane and in the intercellular spaces of palisade tissues and vascular bundles (Fig. S1).

The malondialdehyde (MDA) content in the flag leaf followed a pattern similar to that of ROS in Nn and Ne treatments (Fig. 1a,b). In the excessive N treatment, MDA content increased during the period from anthesis to early dough, indicating that excess N might cause serious cell membrane lipid peroxidation.

### Hyperspectral vegetation index

NDVI is one of the most widely used NIR-based vegetation indexes. From anthesis to maturity, NDVI values continually decreased. No significant difference in NDVI values was observed between Nn and Ne treatments until the early dough stage; however, at the hard dough stage, the vegetation index decreased more rapidly and was significantly lower in the Ne than in the Nn treatment (Fig. 1c).

### Activity of antioxidant enzymes

**Figure 2:**
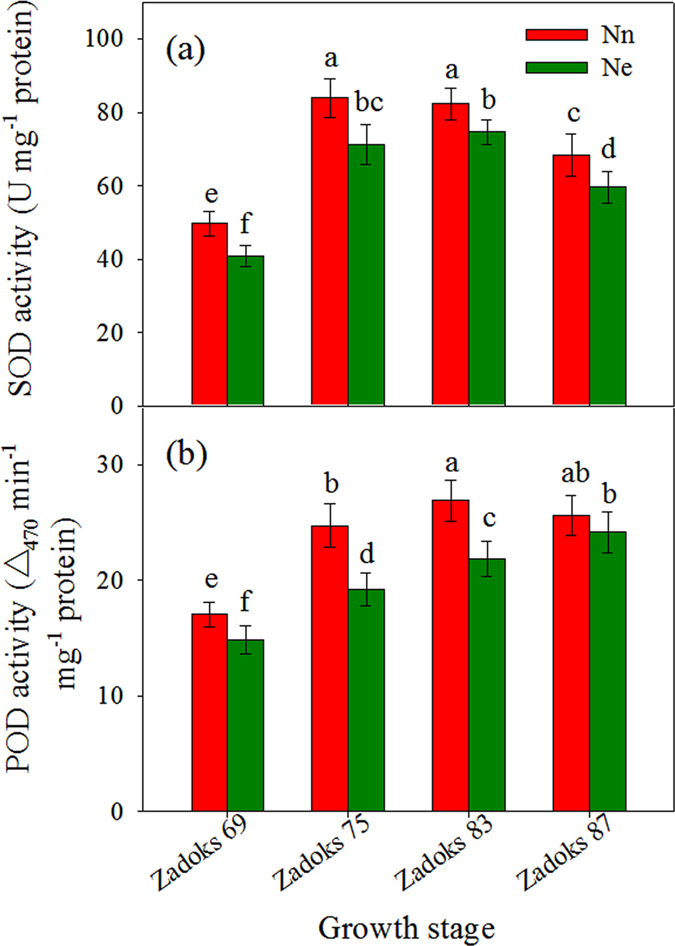
Comparison of the activities of SOD (**a**) and POD (**b**) in flag leaf from the Nn and Ne treatments. Each value represents the mean ± SD from four independent samples. The columns labeled with different letters are significantly different at *p* < 0.05 according to Duncan’s test for multiple comparisons using DPS software.

Actions of SOD and POD are the primary defensive barriers against ROS, and these enzymes are considered antioxidant response markers. In this study, we found that the activity of flag leaf enzymes increased from anthesis to medium milk (SODs) or to early dough (PODs) stages and then decreased. The activities of both enzymes were significantly depressed by Ne treatment compared with those under Nn conditions throughout the grain-filling phase (Fig. 2a,b).

### Metabolite profiling of the response of flag leaves to Ne treatment

In the present study, LC/MS analysis of the leaf metabolome was performed using plants at the early dough stage grown at normal and excessive N supply. The results showed that 99 metabolites of flag leaf were significantly affected by the Ne treatment (Table 1). The concentration of some amino acids (AA) and AA-derived N-acetylleucine and L-lysopine increased strongly under Ne conditions, whereas other N-containing molecules, such as γ-aminobutyric acid (GABA), choline, acetylpyrrolidine and (4-hydroxybenzoyl) choline, decreased. Many organic acids were significantly affected by the treatment, including 2-pyrrolidineacetic acid, coumaric acid, 4-isopropylbenzoic acid, 2′-deoxymugineic acid, citramalic acid, indoleacrylic acid, 6-pentadecyl salicylic acid, and salicylic acid. Except for uracil, uridine and adenosine, most of the bases and nucleosides, including 5-methylcytosine, thymine, adenine, guanine, thymidine and guanosine, increased, indicating that DNA and RNA replication might be affected, and thereby influencing the translation of proteins (enzymes). Two classes of glycerides and seven classes of phospholipids in extracts from wheat leaves were detected by LC-MS (Table 1). The glycerides were monoacylglyceride (MG) and diacylglyceride (DG), and the phospholipids were phosphatidylcholine (PC), phosphatidylserine (PS), phosphatidylinositol (PI), phosphoglycerol (PG), phosphatidic acid (PA) and two types of lysophospholipids. However, the content of most of the glyceride compounds decreased in the Ne treatment. The content of diterpenes, including abietol, serratol and copalic acid, increased, whereas different effects were observed for the contents of triterpenes (ursolic acid, lucidenic acid G and ganoderic acid). The content of most flavonoids (*p*-coumaroyl quinic acid and pachypodol acid), flavonol glycosides (licoagroside A, graveobioside B and keioside) and phenolic compounds (coumarin, kaempferide, *m*-coumaric acid/*p*-coumaric acid and syringic acid) decreased. Alkaloid content (4-hydroxyphenylacetaldehyde and nicotinic acid) also decreased.

Metabolic pathways were constructed to show the influence of Ne treatment on the metabolites involved in N and C metabolism at the early dough stage. The contents of serine, proline, glutamine, lysine, L-methionine, phenylalanine, arginine, tyrosine and glyceric acid were up-regulated in Ne leaves (Table 1).

**Figure 3:**
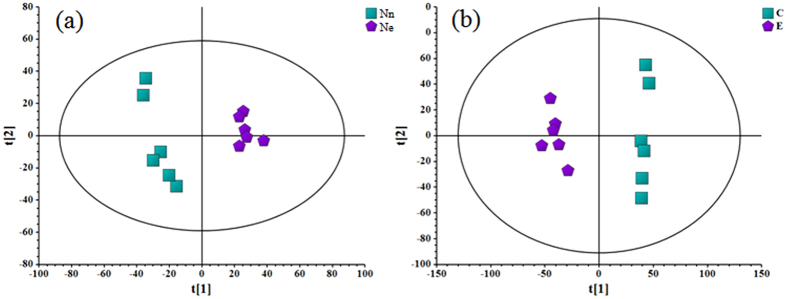
Score plots of PCA of the metabolites in wheat from Nn and Ne treatments with 6 biological replicates. The two groups are clearly separated. In the score plots, each data point represents one flag leaf sample of wheat, and the distance between points indicates the similarity between samples. (**a**) ESI+; (**b**) ESI−. [t1] and [t2] represent PC1 and PC2, respectively.

Multivariate analysis of the datasets using a PCA modeling method revealed a clear separation of metabolite samples between the control and high-N treatment groups (R2X = 0.751, Q2 = 0.483 in ESI+ mode; R2X = 0.765, Q2 = 0.501 in ESI− mode) (Fig. 3a,b), indicating that N fertilizer played a critical role in the metabolic activity of flag leaves.

**Figure 4:**
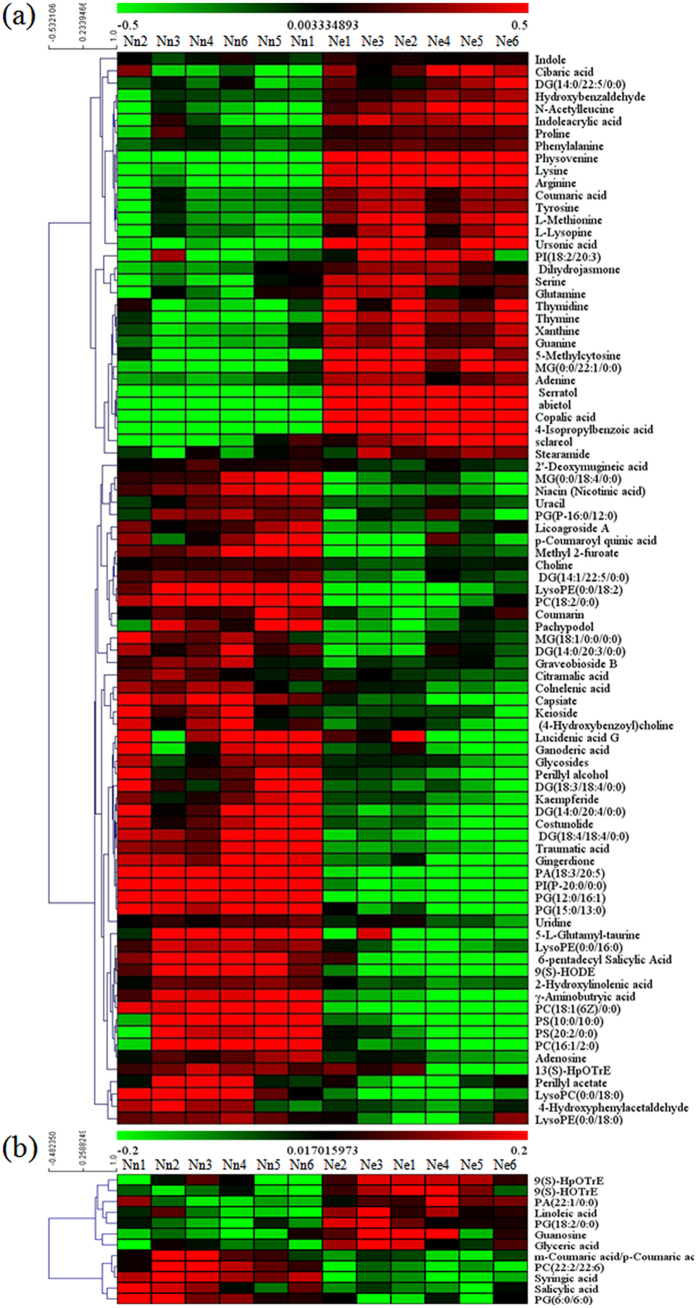
Clustering analysis of metabolomic data in wheat at the early dough stage in Nn and Ne groups. A heat map representation of the levels of 87 metabolites in ESI+ (**a**) and 12 in ESI− mode (**b**) from flag leaves. Two classes are shown in ESI+, and three classes are shown in ESI−. Each line in the heat map represents a metabolite. Red indicates metabolite levels greater than the median value, and green indicates metabolite levels lower than the median value.

The metabolite expression patterns at the early dough stage are shown in a heat map (Fig. 4). Both groups showed two primary patterns in ESI+ mode. Pattern I included 33 metabolites for which expression was up-regulated in high-N treatment group, whereas the other 54 metabolites were down-regulated in pattern II (Fig. 4a). Two primary patterns were shown in both groups in ESI−mode. Pattern I included 7 metabolites that were up-regulated in high-N treatment group; pattern II included 5 metabolites that showed a strong decrease in expression due to high N treatment (Fig. 4b).

### C/N ratio and grain filling

The effects of the two N rates on the C/N ratio are shown in Table 2. The Ne treatment had no significant effect on organic C accumulation in the flag leaves during the anthesis and early dough stages, but the organic C content decreased significantly at the hard dough stage. Flag leaf N content decreased consistently in both N treatments after anthesis. In the Ne treatment at anthesis, the total N content of flag leaves decreased significantly compared with the Nn treatment. However, after anthesis, Ne leaves had higher N contents than those in Nn leaves. As a consequence, the Ne leaves had a significantly higher C/N ratio than that in Nn leaves at anthesis (*p* < 0.05) but lower ratios at the medium milk, early and hard dough stages (*p* < 0.05).

The grain mass was significantly higher under Ne conditions at medium milk than that in the Nn treatment but was similar at the early and hard dough stages under both N treatments (Table 2). However, the maximum grain weight decreased significantly in the Ne treatment at the maturity stage compared with that in the Nn treatment. Analysis of the difference in the grain weights among stages showed that the grain-filling rate was similar between medium milk and early dough stages under both N treatments; however, the rate was significantly lower in the Ne treatment in the following phases than that in the Nn treatment.

## Discussion

During grain filling, cereal crops begin senescence with a burst of excessive ROS such as H_2_O_2_, superoxide and its more toxic derivative hydroxyl radical^25^,^26^. Although N is an essential component of the proteins used to build cell materials and plant tissues, high levels of N are toxic to plant growth^2^. The toxicity of NH_4_^+^ is likely caused by oxidative stress from the excessive accumulation of ROS^26^,^27^,^28^,^29^,^30^. To control such oxidative stress, plants developed numerous strategies for the detoxification of ROS. Among antioxidative enzymes, SODs and PODs play key roles in ROS detoxification in cells. In this study, urea, which is the most commonly used source of organic N, was used as the N fertilizer. We observed that the levels of two antioxidant enzymes (SODs and PODs) decreased in the leaves of wheat under an excessive N conditioncompared with those in wheat treated with an appropriate level of N. Simultaneously, the ROS levels increased as the activity of the antioxidant enzymes declined in the Ne treatmentcompared with those in the Nn treatment (Figs 1 and 2). ROS, detected cytochemically as cerium perhydroxide precipitate, were primarily localized in the cell membrane and cell wall (Fig. S1). Therefore, based on these results, we concluded that excessive N inhibited the activities of ROS scavenging enzymes, which resulted in increased oxidative stress.

Some evidence suggests that the overproduction of ROS can cause a series of oxidative damages to proteins, lipids and DNA, resulting in lipid peroxidation, cellular damage and cell death^8^,^26^,^31^,^32^,^33^. The level of MDA is used routinely to indicate the extent of lipid peroxidation in leaves. Accompanied by the increase in the ROS, MDA contents increased significantly in the Ne treatment. Lipid peroxidation primarily occurs through the action of ROS and is a well-defined marker of oxidative stress in cells. Thus, our results indicated that wheat plants suffered from a greater degree of oxidative damage under the Ne treatment than that in the Nn treatment, which might cause earlier senescence in wheat.

Ground-based spectral radiometers are commonly used to identify relationships between vegetation indexes and canopy N accumulation and crop yield^34^ and senescence progress^22^. NDVI is an easy and nondestructive methodology to characterize genetic resources in response to abiotic stresses^21^. In the present study, NDVI values were similar between the anthesis and early dough stages for the two levels of N; however, the NDVI value was significantly lower in the Ne treatment than that in the Nn treatment at the hard dough stage, indicating that senescence might occur earlier with excessive N application, possibly as aresult of the increased accumulationof ROS.

To analyze metabolic changes, we used a LC/MS technique to profile the metabolites of flag leaves in the two N treatments. Significant metabolite differences were observed between Ne and Nn treatments. Notably, many effective ROS-scavenging metabolites including GABA, 4-hydroxybenzoyl-choline, costunolide, 5-L-glutamyl-taurine, kaempferide, capsiate, licoagroside A, tsugaric acid B and several phenolic compounds were significantly down-regulated in leaves under excessive N conditions. GABA is a nonprotein amino acid that may be a signaling molecule in plants involved in regulating numerous stress response mechanisms such as osmotic potential, heat tolerance, ROS scavenging, pH regulation, energy production and maintenance of the C/N balance^35^,^36^,^37^,^38^. GABA also likely provides partial protection to plants from stresses by elevating leaf turgor by increasing the accumulation of osmolytes and reducing oxidative damage by stimulating enzymatic and nonenzymatic antioxidants in rice^38^, foxtail millet^19^ and wheat^39^. During senescence, the content of GABA increases strongly and is correlated with an increase in glutamate decarboxylase GAD2 gene expression^40^, a process that is essential for nutrient remobilization.

One of the hydroxy radical scavengers in white mustard (*Sinapis alba*)^41^ is 4-hydroxybenzoyl-choline, and costunolide acts as a potential antioxidant by significantly increasing SOD, catalase and GPx activity^42^. 5-L-glutamyl-taurine is associated with the metabolism of taurine, which has antioxidant activity^43^, and kaempferide is a product of genes responsible for antioxidant properties^44^. The antioxidant activity of synthesized capsiates was determined and confirmed using *in vitro* assays^45^, and these capsiates show a concentration-dependent ROS scavenging capacity that neutralizes superoxide radicals^46^. Licoagroside A has antioxidative ability against ROS generation in the skin^47^, and tsugaric acid B presents antioxidant activity in Ganoderma^48^. However, in the Ne treatment, these compounds all decreased. Additionally, most of the phenolic and flavonoid compounds were down-regulated in the Ne treatment in this study (Table 1). Phenolic and flavonoid compounds are widely acknowledged to function as key antioxidants in stressed plants by scavenging ROS and by inhibiting bursts of ROS^49^,^50^. Compounds derived from proline and pyrrolidine-2-acetic acid inhibit GABA uptake and transport^51^,^52^; however, proline and pyrrolidine-2-acetic acid were also up-regulated in Ne treated plants (Table 1).

Linoleic acid, a polyunsaturated fatty acid within cell membranes, is oxidized by singlet oxygen via the function of 9-LOX (lipoxygenase), which leads to the synthesis of the fatty acid hydroperoxide, 9(S)-HODE (9-hydroxy-10,12(E,Z)-octadecadienoic acid)^53^,^54^. This process may be a mechanism to deplete ROS and produce 9(S)-HODE as a plant defense strategy^54^. However, in the Ne treatment, the increase in the accumulation of linoleic acid and the decrease in the level of 9(S)-HODE indicated that the autoxidation of linoleic acid might have decreased, most likely due to reduced 9-LOX activity. More importantly, our LC/MS analysis showed that most of the cell membrane localized lipids, which were specifically identified in this study, were down-regulated under Ne conditions. Phosphatidic acid (PA) plays a major role in plant growth and development and is involved in regulating root hair growth. PA is rapidly and transiently generated in response to biotic and abiotic stress in plants, including drought, salinity, pathogen attack and heat stress. Heat stress induces a rapid increase in the second messenger PA, which is responsible for an influx of Ca^2+^ and in turn modulates the expression of heat-shock proteins^55^,^56^. PA is also involved in membrane trafficking and cellular reorganization during salt stress^57^. Diacylglycerol (DG) is a well-known signaling lipid in animals. In plants, DG can be further phosphorylated by DG kinases to generate PA^58^, but its role in plants remains elusive. Similarly, PI is an important signaling molecule in cellular processes, such as membrane trafficking, cytoskeleton organization, polar tip growth and stress responses^57^. PC, PS, PG, and PI are important lipids that function as integral parts of thylakoid membranes^57^,^59^. Maintenance of lipid composition may be used as a strategy in species with high resistance to long-term moderate heat, whereas species with less resistance show lipid degradation^60^. Lysophospholipids are minor membrane components and signaling mediators in numerous tissues and are relevant stress markers in plants^57^.

Because these ROS scavenging metabolites included secondary metabolites such as flavonoids and phenolics and amino acid derivatives such as GABA and lipids, we hypothesized that the strong disturbance to N metabolism, secondary metabolism and lipid metabolism caused by high N application was attributed to the depletion of ROS scavenging metabolites.

In addition to showing activity neutralizing the adverse effects of salt stress by activation of genes encoding SOD to scavenge ROS^61^, traumatic acid and the related metabolite 2-hydroxylinolenic acid are often described as markers of quantitative resistance to Fusarium Head Blight^18^,^62^. *Nicotiana attenuata* is protected from attack by *Manduca sexta* larvae^63^ by 2-hydroxylinolenic acid, and fungal growth is inhibited by colnelenic acid^64^. Salicylic acid is a key compound in establishing systemic acquired resistance and induces the expression of the pathogenesis-related protein PR1b in barley^17^. Antibacterial activity against *Streptococcusmutans* and *Porphyromonas gingivalis* is shown by 6-pentadecyl salicylic acid^65^. However, these metabolites were all down-regulated in our LC/MS analysis (Table 1), which strongly supported previous observations that high N increases plant disease susceptibility^3^,^4^.

Heat, drought and pathogen infection are the most important environmental stresses limiting the growth and productivity of wheat during the grain-filling phase in northern China, and to defend against these stresses, wheat requires strong antioxidant capability. However, based on this study, we propose that excessive N treatment decreased the antioxidative ability of wheat because cell membrane-localized lipids were generally down-regulated, leading to the increased production of ROS and then the peroxidation of membrane lipids as indicated by the increased level of MDA.

Metabolite analysis showed less accumulation of many compounds such as nicotinic acid, choline, citramalic acid, 2′-deoxymugineic acid and salicylic acid (Table 1). Nicotinic acid is one of the biologically active substances^66^. Choline is converted to betaine aldehyde by choline monooxygenase, which is then converted to glycinebetaine by betaine aldehyde dehydrogenase^67^. Growth increases in transgenic rice overexpressing choline monooxygenase^68^. Additionally, choline functions as a component of amino acid transporters. Citramalic acid and salicylic acid in sugar beet root exudates solubilize soil phosphorus^69^. A metal chelator in leaves, 2′-deoxymugineic acid may be relevant for the mobilization and retranslocation of metals in high-yielding wheat production^70^. Increasing concentrations of 2′-deoxymugineic acid by transgenic techniques increases grain Zn and Fe concentrations^71^,^72^. In older leaf tissues, salicylic acid accumulates, resulting in the activation of autophagy^73^, which has an important role in N remobilization by contributing to the dismantling of chloroplasts during senescence. In salicylic acid-deficient autophagy mutants (*atg5.sid2*), the autophagy machinery is defective, and the double mutants are inefficient in the remobilization of N^74^. By contrast, *p*-hydroxybenzaldehyde increased in flag leaves under excessive N treatment based on our LC/MS measurement. This compound completely inhibits seed germination in wheat and radish (*Raphanus sativu*), inhibits shoot and root elongation in wheat^75^ and contributes to the phytotoxicity of common nandina (*Nandina domestica* Thunb.) in four plants including wheat^76^.

Generally, heat stress causes oxidative damages to plant cells and in foxtail millet, leads to the accumulation of amino acids^19^. In the present study, eight amino acids were significantly up-regulated in the excessive N fertilizer treatment at the early dough stage, suggesting that N remobilization and/or other physiological processes might be affected. The increase in the contents of proline, glutamine and arginine indicated that the activity of the urea cycle was higher in leaves of the Ne treatment than that in Nn leaves^77^. The increase in the accumulation of serine, glyceric acid, phenylalanine and tyrosine and the decrease in the accumulation of *p*-coumaric acid in leaves of the excessive N treatment suggested that carbon metabolism was disrupted^77^. In a previous study, increases in methionine and lysine indicated that the conversion of aspartate to amino acids remained active^77^. With an accumulation of phenylalanine and tyrosine (two metabolites involved in the primary steps of the phenylpropanoid pathway), lignin biosynthesis is altered, which possibly affects ear development^16^. In the present study, we found an increase in the accumulation of phenylalanine and tyrosine (Table 1; Fig. S2), suggesting a decrease in lignin biosynthesis. These results are highly consistent with our earlier observation that high N decreases lignin content and thus decreases the culm mechanical strength and grain-filling rate^2^.

The C-N balance is important for plant function. The C/N ratio is closely associated with autophagy in *atg* mutants and bulk protein degradation during leaf senescence (partially due to the regulation of transcription of a C1A cysteine protease)^78^ and is likely a good indicator of plant growth, senescence and nutrient remobilization^14^,^79^. In the present study, the C/N ratio increased significantly in the Ne treatment compared with that in the Nn treatment at anthesis, but contrasting results followed. Simultaneously, we found a parallel change in the grain-filling rate, namely, the grain-filling rate increased during anthesis and the medium milk stage and then subsequently decreased in the following stages in the Ne treatment. An excessive supply of N does not increase wheat growth and yield or fertilizer recovery^80^,^81^ and reduces chlorophyll content, net photosynthetic rate, grain-filling rate and grain yield^82^,^83^,^84^,^85^,^86^. Therefore, based on our results, we propose that the Ne treatment causes oxidative stress to wheat. Because oxidative stress affects the autophagy pathway^74^, the accumulation of carbohydrates and remobilization of N were affected, and as a result, the C/N ratio decreased. Indeed, a recent research suggests that heat-stress induced oxidative damages post-anthesis is closely associated with a reduction in the C/N ratio in the flag leaves of wheat^87^. Therefore, N fertilizer use that is based on plant requirements is crucial for the highly efficient use of N.

In conclusion, according to the physiological evidence, excessive N significantly decreased the activity of SODs and PODs. Additionally, the comparative metabolomic analysis revealed less accumulation of many nonenzymatic antioxidants that are involved primarily in oxidative scavenging. As a consequence, ROS and MDA contents increased, and NDVI values for the canopy decreased much more rapidly in the excessive N treatment compared with the normal N-treated flag leaves. The comparative metabolomic analysis also indicated less N remobilization, lower resistance to pathogen attacks, and decreased resistance to heat stress in the excess N treatment compared with the normal-N fertilizer treatment. The C/N ratio was altered by excessive N because N metabolism and remobilization were disrupted, in addition to effects on carbohydrate accumulation in the later stages. Therefore, with the application of excessive N, we conclude that oxidative stress increased partly due to the inactivation of antioxidant enzymes and a decrease in ROS scavenging metabolites; in addition, N metabolism, secondary metabolism and lipid metabolism were disturbed; as a result of these changes, leaf senescence accelerated, and the grain-filling rate and ultimately grain yields in wheat were reduced.

## Methods

### Plant material and growth conditions

Winter wheat Jimai 22, a widely grown cultivar in China, was planted in 2014–2015 in a field at an experimental station (36°42′N, 117°4′E; altitude 48 m) of the Shandong Academy of Agricultural Sciences, China. The soil type is classified as sandy loam (pH 7.4). The top 40 cm of soil contained 67.3 mg kg^−1^ water-hydrolyzable N, 22.1 mg kg^−1^ rapidly available phosphorous, 139.3 mg kg^−1^ rapidly available potassium and 1.84% organic matter. The sowing date was October 7, 2014. The plot size was 2.0 × 10.0 m^2^; there were ten rows per plot 20 cm apart. Before planting, 10 g m^−2^ P_2_O_5_, 10 g K_2_O m^−2^ and 7.5 g N m^−2^ were applied to the soil. At the shooting stage (Zadoks stage 31), 15 g N m^−2^ was top-dressed as the normal N treatment (Nn), and 30 g N m^−2^ was applied as the excessive N treatment (Ne). All N fertilizer was applied as urea (NH_2_)_2_CO. All of the analyses were conducted during grain filling.

### Determination of ROS

The ROS concentration was determined using DCFH-DA (Sigma-Aldrich, Germany) as described by Kong *et al*.^5^ with slight modifications. A 25 mM solution of DCFH-DA was prepared in dimethyl sulfoxide and stored at −20 °C until use. Sections cut from the flag leaves (*ca.* 0.2 mg) were washed with 50 mM methyl ethanesulfonate buffer (pH6.2) and transferred to 100 μl of fresh buffer in small wells of ELISA plates containing 10 μM DCFH-DA. Following incubation at 25 °C in the dark for 20 min, the fluorescence was immediately measured with an excitation wavelength of 485 nm and an emission wavelength of 535 nm using an ELISA plate reader (GENios Pro, Tecan, Switzerland). The ROS concentration units were defined as the average increase in the DCF fluorescence per mg of fresh sample per minute.

### Localization of hydrogen peroxide (H_2_O_2_)

The active oxygen species H_2_O_2_ was detected cytochemically by its reaction with cerium chloride (CeCl_3_) to produce electron-dense deposits of cerium perhydroxides. Tissue pieces (approximately1 mm^3^) were excised from flag leaf of wheat under normal or excessive N treatments and were immediately perfused in 10 mM CeCl_3_ (Sigma) in 50 mM 3-morpholinopropanesulfonic acid (MOPS) (pH7.2) for 1 h. Tissues were then fixed in 2.5% glutaraldehyde in 0.1 M sodium cacodylate (CAB) buffer (pH7.2) for 4–24 h. After fixation, tissues were washed twice for 10 min in CAB buffer and postfixed for 4 h in 1% osmium tetroxide in 0.1 M CAB. Tissues were dehydrated in an ethanol series at room temperature and were finally embedded in epon resin 812 at 60 °C. Ultrathin sections (70 to 90 nm) stained with 2% aqueous uranyl acetate for 10 min were examined using a transmission electron microscope (JEM-1400; JEOL, Tokyo, Japan) at an accelerating voltage of 75 kV. H_2_O_2_was localized as electron-dense precipitates of cerium perhydroxides^88^.

### Assessment of lipid peroxidation

The MDA content was measured according to the methodology from Dhindsa and Matowe (1981)^89^ with a slight modification. Plant tissue (approximately 0.50 g) was homogenized in 5 ml of trichloroacetic acid (10%) and centrifuged at 10,000 × g for 15 min. To a 1-ml aliquot of the supernatant, 4 ml of 0.5% TBA was added, and the mixture was heated at 95 °C for 30 min, quickly cooled, and then centrifuged at 10,000 × g for 10 min. The absorbance was read at 450, 532, and 600 nm, and MDA content was calculated according to the following equation: MDA = 6.45 × (A532−A600)−0.56 × A450.

### Measurement of the hyperspectral vegetation indexes

The normalized difference vegetative index (NDVI) was determined for wheat in the Nn and Ne treatments using a portable spectroradiometer (GreenSeeker handheld crop sensor; Trimble, USA). The sensor was held 60 cm above the canopy. NDVI was calculated from measurements of light reflectance in the red and near-infrared (NIR) regions of the spectrum as follows: (NIR − R)/(NIR + R), where R is the reflectance in the red band and NIR is the reflectance in the near-infrared band.

### Enzymatic antioxidant activity

For determinations of the antioxidant enzyme activities, enzyme extracts were prepared by grinding the leaf samples (0.5 g) in liquid N, followed by homogenizing the powder in 10 ml of 50 mM SPB (pH7.0) containing 1 mM EDTA and 1% polyvinylpyrrolidone (PVP-40). The homogenate was centrifuged for 20 min at 15,000 × g. The supernatant was used to determine the activities of peroxidase (POD, EC 1.11.1.7) and superoxide dismutase (SOD, EC 1.15.1.1). SOD activity was assayed by monitoring superoxide radical-induced nitroblue tetrazolium reduction at 560 nm, and one unit of SOD activity was defined as the amount of enzyme that caused 50% inhibition of this reaction in comparison with a blank sample. POD activity was determined using the guaiacol oxidation method. One unit of POD activity was defined as the change in absorbance per minute and specific activity as enzyme units per gram of fresh sample. Proteins were quantified by the protein dye binding method of Bradford (1976)^90^, using bovine serum albumin (BSA) as the standard.

### LC/MS analysis

Flag leaves were sampled from the plants at the grain-filling stage. Collected samples were flash frozen and stored at −80 °C until metabolic analysis using an established LC-MS-based approach^91^. Chromatographic separation was performed using an Orbitrap Elite mass spectrometer (Thermo Scientific Inc., Bremen, Germany) coupled with a Dionex UltiMate 3000 UHPLC system (Thermo Scientific Inc., Bremen, Germany) and equipped with a C18 reversed-phase column (Hypergod; 100 mm × 4.6 mm × 3 μm). The column was maintained at 40 °C. The injected sample volume was 4 μl. The metabolites were eluted with a gradient of 95% A plus 5% B for 0–12 min, 5% A plus 95% B for 2–17 min, and 95% A plus 5% B for 17–19 min. Solvent A was water with 0.1% formic acid, and solvent B was acetonitrile with 0.1% formic acid. The flow rate was 0.3 ml/min.

All sample extracts were analyzed in both positive ion mode (ESI+) and negative ion mode (ESI−) for six replicates. For ESI+ acquisition, instrumental settings were optimized to maximize the signal with the final parameters as follow: heater temp 300 °C, sheath gas flow rate 45 (arbitrary units), aux gas flow rate 15 (arbitrary units), sweep gas flow rate 1 (arbitrary units), spray voltage 3.0 kV, capillary temperature 350 °C and S-lens RF level 30%. ESI-acquisition was conducted with the following differences: spray voltage 3.2 kV and S-lens RF level 60%. The metabolites that differed between the two classes were quantified using a combination of VIP statistics (threshold >1) of the OPLS-DA model and *t*-tests (*p* < 0.05). Compounds were identified by a comparison of m/z or precise molecular mass at http://metlin.scripps.edu.

### Data Processing

The different metabolites from Nn and Ne flag leaves of wheat were identified based on their respective VIP (Variable Importance in the Projection) value, *p*-value and peak intensity for fragmentation. Detected compounds were confirmed using the XCMS informatics platform (http://metlin.scripps.edu/) and running pure analytical standards.

Following analysis in XCMS Online, the data were mean-centered and Pareto-scaled and exported to Simca-P (version 13.0; Umetrics, Umea, Sweden) for multivariate analysis. All spectral data were processed using the software SIEVE v.2.0, normalized and organized into a two dimension matrix including retention time and mass to charge (m/z) ratio and peak intensity. The matrix of molecular features was analyzed using principal components analysis (PCA) to determine the differences in metabolite profiles between the two groups. Following PCA analysis, all data were mean-centered and univariate-scaled and divided into two groups: Nn and Ne,b efore analysis by Orthogonal Projections to Latent Structures via partial least-squares-Discriminant Analysis (OPLS-DA).

Fold change for each metabolite identified by LC-MS analyses of the tissue samples was calculated as the mean relative quantity of that compound in the excessive N treatment divided by the quantity in the control N treatment. To visualize the entire data set, the web-based software Metaboanalyst was used to construct a heat map using a hierarchical clustering method and to perform the *t*-test and calculate the *p*-value and Log2 fold change for individual metabolites. Correlations between the important metabolites and inflammatory cells were calculated using Pearson’s correlation analysis built in Metaboanalyst 2.0. The metabolic pathway was referred to the KEGG database.

### Determination of organic C and total N

Flag leaves were collected at anthesis, medium milk, early dough (Zadoks 83) and hard dough stages and oven-dried to a constant weightat 60 °C. The samples were pulverized and milled (<0.20 mm) for the measurement of organic C and N content. Organic C was determined using the K_2_Cr_2_O_7_-H_2_SO_4_ wet oxidation method^92^. To determine the total N, the samples were first mineralized using the Kjeldahl method. The total N content was measured using an automatic N analyzer (BUCHI AutoKjeldahl Unit K-370; BUCHI Laboratory Equipment, Flawil, Switzerland).

### Grain weight

In all subplots in each treatment, a small plot combine was used to harvest the grain from the subplot area of 6 m^2^ when the plants reached physiological maturity. The grains were air-dried to 13% moisture and weighed.

### Statistical Analyses

One-way analysis of variance (ANOVA) was conducted for each parameter studied using the Data Processing System (DPS) statistical software (v.14.10, Refine Information Tech. Co., Ltd., Hangzhou, Zhejiang, China)^93^. Duncan’s multiple range tests were used to evaluate the statistical significance of the results. The data are presented as the means ± SD, and significant differences among the treatments are shown with different letters. A general linear model ANOVA was used for the statistical analysis of the mixed effects of the N levels and the date of sampling on ROS concentration, MDA, antioxidative enzymes, and other physiological indices.

## Additional Information

**How to cite this article**: Kong, L. *et al*. Excessive nitrogen application dampens antioxidant capacity and grain filling in wheat as revealed by metabolic and physiological analyses. *Sci. Rep.* **7**, 43363; doi: 10.1038/srep43363 (2017).

**Publisher's note:** Springer Nature remains neutral with regard to jurisdictional claims in published maps and institutional affiliations.

## Supplementary Material

Supplementary Information

## Figures and Tables

**Table 1:** Metabolite comparison with significant differences between excessive N (Ne) treatment and control in both ESI+ and ESI− modes.

	VIP	Metabolite	Detected mass	RT (min)	*t*-test	fold change (Ne/Nn)
ESI+	1.511	γ-Aminobutyric acid	103.0632	1.00	0.000	−0.901
	1.414	Choline	103.0993	0.95	0.000	−0.211
	1.395	Serine	105.0425	0.91	0.000	0.686
	1.142	Uracil	112.0269	1.01	0.009	−0.252
	1.223	Proline	115.0631	1.03	0.003	0.304
	1.070	Indole	117.0577	5.36	0.017	0.099
	1.442	Hydroxybenzaldehyde	122.0366	1.04	0.000	0.432
	1.456	Niacin (Nicotinic acid)	123.0317	1.21	0.000	−0.786
	1.464	5-Methylcytosine	125.0586	2.73	0.000	1.104
	1.395	Methyl 2-furoate	126.0313	1.11	0.000	−0.849
	1.500	Thymine	126.0427	3.87	0.000	0.953
	1.109	2-Pyrrolidineacetic acid	129.0786	0.98	0.012	1.190
	1.455	Adenine	135.0543	3.46	0.000	0.516
	1.008	4-Hydroxyphenylacetaldehyde	136.0522	4.56	0.027	−0.370
	1.096	Coumarin	146.0364	4.20	0.013	−0.408
	1.071	Glutamine	146.0690	0.92	0.016	0.409
	1.563	Lysine	146.1053	0.85	0.000	1.265
	1.217	Citramalic acid	148.0373	4.78	0.004	−0.261
	1.496	L-Methionine	149.0507	1.62	0.000	0.811
	1.489	Guanine	151.0493	3.82	0.000	0.633
	1.452	Xanthine	152.0332	3.82	0.000	0.577
	1.217	Perillyl alcohol	152.1199	5.44	0.004	−0.702
	1.477	Coumaric acid	164.0471	2.32	0.000	0.567
	1.563	4-Isopropylbenzoic acid	164.0833	10.76	0.000	1.341
	1.506	Phenylalanine	165.0785	3.90	0.000	0.345
	1.283	Dihydrojasmone	166.1354	4.17	0.002	0.389
	1.361	N-Acetylleucine	173.1049	4.43	0.000	1.015
	1.558	Arginine	174.1114	0.91	0.000	1.201
	1.483	Tyrosine	181.0736	2.31	0.000	0.578
	1.492	D-Sorbitol	182.0769	2.32	0.000	0.572
	1.438	Indoleacrylic acid	187.0630	4.01	0.000	0.808
	1.008	Perillyl acetate	194.1306	7.56	0.027	−0.512
	1.346	L-Lysopine	218.1262	1.54	0.001	0.603
	1.200	(4-Hydroxybenzoyl) choline	224.1271	3.93	0.005	−0.469
	1.479	Traumatic acid	228.1357	4.57	0.000	−0.784
	1.366	Costunolide	232.1458	5.47	0.000	-0.784
	1.320	Thymidine	242.0899	3.90	0.001	0.686
	1.154	Uridine	244.0690	2.29	0.008	-0.223
	1.201	5-L-Glutamyl-taurine	254.0575	6.38	0.005	−2.068
	1.549	Physovenine	262.1312	4.07	0.000	1.585
	1.286	Adenosine	267.0961	3.50	0.001	−0.377
	1.286	Dibutyl phthalate	278.1514	8.85	0.001	0.259
	1.522	Abietol	288.2449	4.72	0.000	2.682
	1.562	Serratol	290.2605	4.93	0.000	2.033
	1.096	Colnelenic acid	292.2033	6.41	0.013	−0.348
	1.405	2-Hydroxylinolenic acid	294.2189	5.60	0.000	−0.362
	1.418	9(S)-HODE	296.2346	7.29	0.000	−1.168
	1.272	Kaempferide	300.0626	4.34	0.002	−0.492
	1.142	2′-Deoxymugineic acid	304.1270	0.76	0.009	−0.143
	1.446	Copalic acid	304.2399	4.38	0.000	2.209
	1.427	Capsiate	306.1825	5.09	0.000	−0.878
	1.306	Sclareol	308.2710	5.19	0.001	0.823
	1.003	13(S)-HpOTrE	310.2139	6.20	0.028	-0.436
	1.258	Cibaric acid	324.1933	6.74	0.002	0.626
	1.031	*p*-Coumaroyl quinic acid	338.0994	4.08	0.023	−0.890
	1.167	Pachypodol	344.0887	4.69	0.007	−0.599
	1.479	Gingerdione	348.2292	5.52	0.000	−0.797
	1.420	6-Pentadecyl salicylic acid	348.2659	9.20	0.000	−1.036
	1.288	MG(0:0/18:4(6Z,9Z,12Z,15Z)/0:0)	350.2448	5.82	0.001	−0.689
	1.174	MG(18:1(11Z)/0:0/0:0)	356.2920	8.62	0.006	−0.520
	1.282	MG(0:0/22:1(13Z)/0:0)	412.3548	10.71	0.002	1.557
	1.454	LysoPE(0:0/16:0)	453.2848	8.57	0.000	−0.625
	1.200	Ursonic acid	454.3438	7.30	0.005	2.425
	1.051	Lucidenic acid G	476.2762	4.65	0.019	−1.286
	1.499	LysoPE(0:0/18:2(9Z,12Z))	477.2845	7.96	0.000	−1.041
	1.102	LysoPE(0:0/18:0)	481.3158	8.37	0.013	−0.405
	1.282	Licoagroside A	492.1253	4.47	0.002	−0.413
	1.462	PC(18:2(9Z,12Z)/0:0)	519.3317	8.26	0.000	−1.224
	1.404	PC(18:1(6Z)/0:0)	521.3470	9.11	0.000	−1.549
	1.373	LysoPC(0:0/18:0)	523.3625	11.03	0.000	−1.250
	1.163	PC(16:1(9Z)/2:0)	535.3266	6.42	0.007	−1.581
	1.062	Ganoderic acid	544.3758	8.25	0.018	−0.968
	1.211	PS(20:2(11Z,14Z)/0:0)	549.3052	5.48	0.004	−0.862
	1.310	PS(10:0/10:0)	567.3161	4.60	0.001	−1.110
	1.174	Glycosides	584.2798	4.97	0.006	−0.363
	1.345	DG(14:0/20:4(5Z,8Z,11Z,14Z)/0:0)	588.4737	12.30	0.001	−1.057
	1.218	DG(14:0/20:3(5Z,8Z,11Z)/0:0)	590.4901	14.99	0.004	−0.489
	1.148	Graveobioside B	594.1570	4.11	0.008	−0.332
	1.310	DG(18:4(6Z,9Z,12Z,15Z)/18:4(6Z,9Z,12Z,15Z)/0:0)	608.4430	8.82	0.001	−1.268
	1.270	DG(18:3(6Z,9Z,12Z)/18:4(6Z,9Z,12Z,15Z)/0:0)	610.4587	10.82	0.002	−0.619
	1.377	PI(P-20:0/0:0)	612.3629	9.04	0.000	−2.266
	1.464	DG(14:1(9Z)/22:5(4Z,7Z,10Z,13Z,16Z)/0:0)	612.4743	12.50	0.000	-0.421
	1.186	Keioside	624.1671	4.18	0.005	−0.465
	1.135	PG(P-16:0/12:0)	650.4501	13.14	0.009	−0.404
	1.366	PG(12:0/16:1(9Z))	664.4294	11.18	0.000	−2.155
	1.281	PG(15:0/13:0)	666.4460	10.97	0.002	-1.776
	1.439	PA(18:3(9Z,12Z,15Z)/20:5(5Z,8Z,11Z,14Z,17Z))	716.4468	7.98	0.000	−2.402
	1.076	PI(18:2(9Z,12Z)/20:3(8Z,11Z,14Z))	884.5436	14.82	0.016	0.863
ESI−	1.063	Glyceric acid	106.0262	1.01	0.014	0.225
	1.119	Salicylic acid	138.0310	3.84	0.008	−0.183
	1.170	*m*-Coumaric acid/*p*-Coumaric acid	164.0467	4.04	0.005	-0.197
	1.461	Syringic acid	198.0518	3.91	0.000	−0.334
	1.178	Linoleic acid	280.2380	8.75	0.004	0.193
	1.109	Guanosine	283.0900	3.74	0.009	0.238
	1.206	9(S)-HOTrE	294.2176	7.75	0.003	0.256
	1.122	9(S)-HpOTrE	310.2115	6.86	0.008	0.249
	1.162	PG(6:0/6:0)	442.2028	4.19	0.005	−0.252
	1.125	PA(22:1(11Z)/0:0)	492.3269	9.00	0.008	0.187
	1.217	PG(18:2(9Z,12Z)/0:0)	508.2855	4.47	0.003	0.191
	1.353	PC(22:2(13Z,16Z)/22:6(4Z,7Z,10Z,13Z,16Z,19Z))	885.6122	0.88	0.000	−0.287

**Table 2:** Effect of excessive N application on flag leaf total N (%), organic C (%), C/N ratio and the grain yield of wheat plants. Each value represents the mean of four replicates ± SE.

Stage	N level	Organic C (%)	Total N (%)	C/N ratio	Grain yield (g m^−2^)	Phase increase (g m^−2^)
Anthesis (Zadoks 69)	Nn	38.23 ± 2.04a	3.91 ± 0.20a	9.78 ± 0.24e	—	—
	Ne	39.59 ± 2.51a	3.61 ± 0.24b	10.97 ± 0.53d	—	—
Medium milk (Zadoks 75)	Nn	39.03 ± 1.51a	3.18 ± 0.15b	12.04 ± 0.55c	127.90 ± 6.01f	127.90 ± 6.01e
	Ne	38.60 ± 1.82a	3.52 ± 0.16c	10.96 ± 0.56d	147.16 ± 4.26e	147.16 ± 4.26d
Early dough (Zadoks 83)	Nn	39.06 ± 2.08a	2.90 ± 0.23d	14.07 ± 0.85b	429.33 ± 11.09d	301.43 ± 10.83a
	Ne	37.15 ± 2.12ab	3.24 ± 0.19c	11.79 ± 0.90cd	444.24 ± 12.66d	297.08 ± 9.99a
Hard dough (Zadoks 87)	Nn	36.72 ± 2.23ab	2.01 ± 0.12f	19.31 ± 0.92a	733.16 ± 8.45c	303.83 ± 12.49d
	Ne	34.00 ± 1.36b	2.36 ± 0.17e	15.74 ± 0.84b	728.66 ± 6.13c	284.42 ± 9.12a
Maturity (Zadoks 92)	Nn	—	—	—	933.52 ± 11.52a	200.36 ± 9.29b
	Ne	—	—	—	905.55 ± 15.53b	176.89 ± 6.39c

Means followed by the same letter within a column are not significantly different at *P* < 0.05.
