# Supplementary material for: Excessive nitrogen application dampens antioxidant capacity and grain filling in wheat as revealed by metabolic and physiological analyses
Source: Sci Rep. 2017 Feb 24;7:43363. doi: 10.1038/srep43363 (PMC5324167; doi:10.1038/srep43363)
Supplement: Supplementary Information [file srep43363-s1.pdf]

Excessive nitrogen application dampens antioxidant capacity and grain filling in wheat as revealed by metabolic and physiological analyses

Lingan Kong[1,]*, Yan Xie[1], Ling Hu[1,2], Jisheng Si[1] & Zongshuai Wang[1]

[1] Crop Research Institute, Shandong Academy of Agricultural Sciences, Jinan 250100, China

[2] College of Life Science, Shandong Normal University, Jinan 250014, China

Running title: Negative effects of excessive N on wheat yield

* **Corresponding author**:

Lingan Kong, Ph.D.

Crop Research Institute

Shandong Academy of Agricultural Sciences

202 Gongyebei Road

Jinan City 250100

China

Tel.: 0086-531-83178123

Fax: 0086-531-83179088

E-mail address: kongling-an@163.com

Supplementary Information

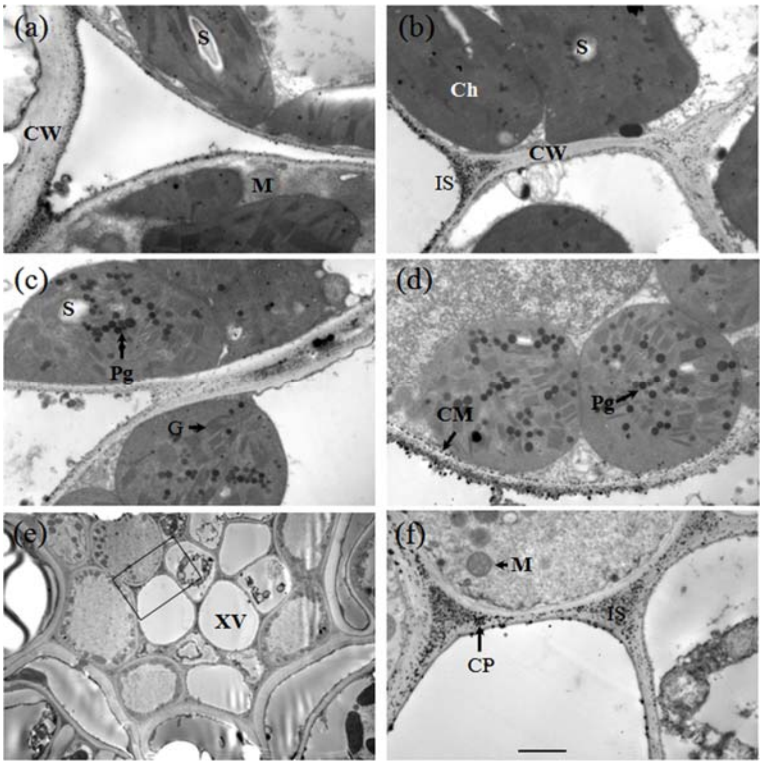

Figure S1. Detection of H_2O_2 in flag leaves of wheat. The presence of H_2O_2 is marked by electron-dense precipitates of cerium perhydroxides, which was localized to cell walls and the cell membrane and intercellular space of palisade tissues and vascular bundle. Bar: (a), (b), (c), (d) and (f): 1 μm; (e): 5 μm. CP, cerium perhydroxides; Ch, chloroplast; CM, cell membrane; IS, intercellular space; M, mitochondria; XV, xylem vessel.

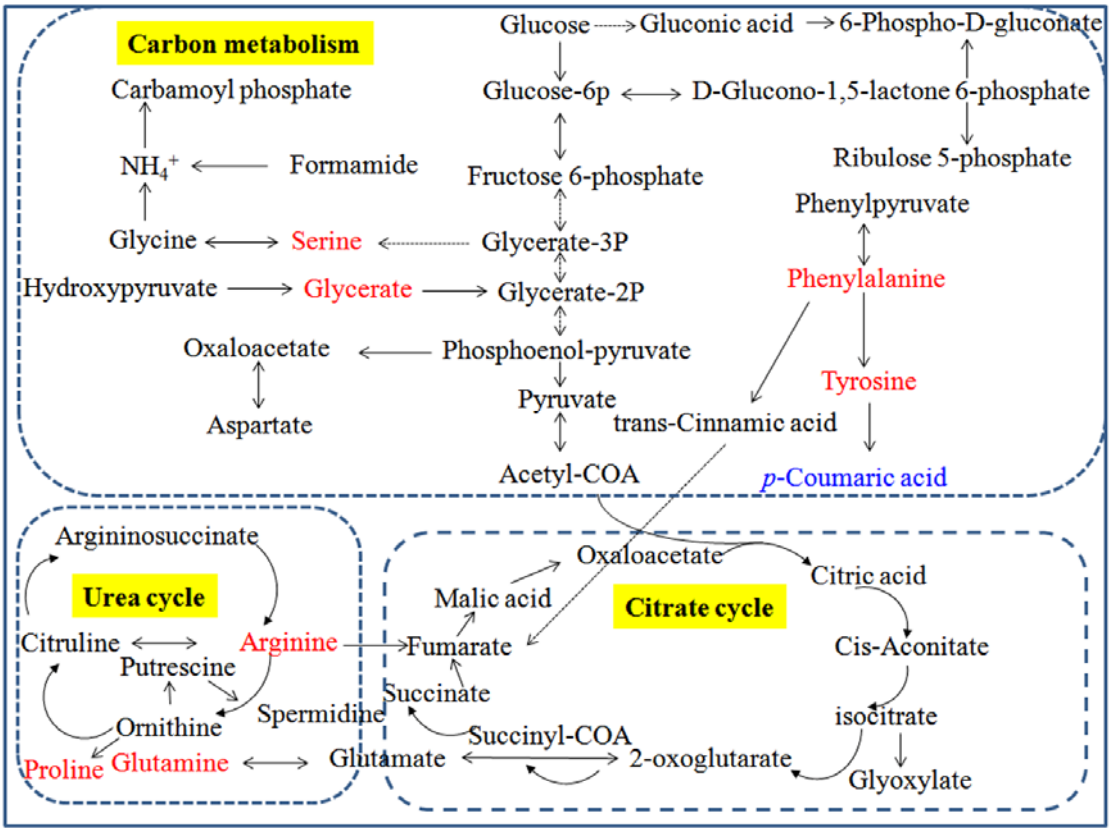

Figure S2. Comprehensive metabolic pathways involved in C metabolism and N metabolism in Nn and Ne flag leaves of wheat. The red font means the up-regulated metabolites for Ne treatment; the blue font means the down-regulated metabolites for Ne treatment.
